# Supplementary material for: Generation of a Novel Nkx6-1 Venus Fusion Reporter Mouse Line
Source: Int J Mol Sci. 2021 Mar 26;22(7):3434. doi: 10.3390/ijms22073434 (PMC8036392; doi:10.3390/ijms22073434)
Supplement: Supplementary file 1 [file ijms-22-03434-s001.pdf]

Supplementary data

Supplementary figure 1. Venus expression marks Nkx6-1-VF expressing cells at embryonic stage. (a) Co-staining of GFP and Nkx6-1 at E18.5 shows co-localization of both markers.

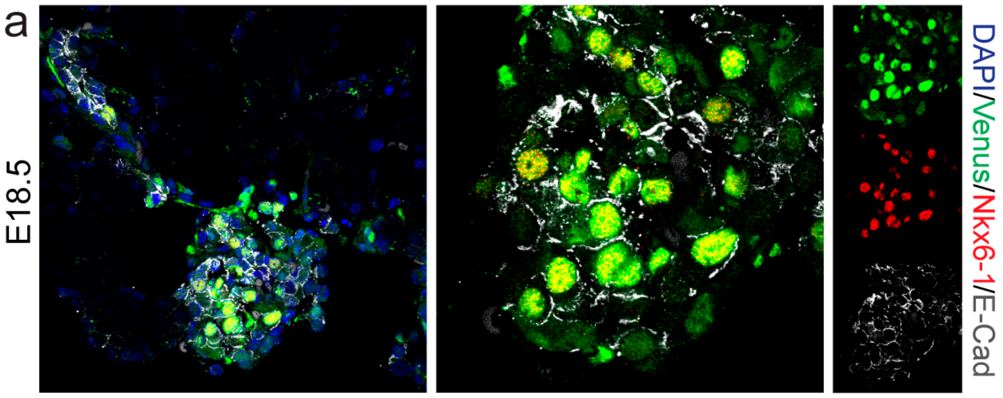

Table 1. Primers sequences

**Primer ID**	**Sequence (5′ – 3′)**
**EP1197**	NNNGCGGCCGCGGGTCTTCCGGCGCTCACCTCCTC
**EP1198**	NNNTCTAGAGGACGAGCCCTCGGCCTCCGAC
**EP1199**	NNNAAGCTTGCGCGACCAGCACCGCGGGGATC
**EP1200**	NNNCTCGAGGACGCCTGACGTCGCGGGTGGG
**EP1126**	GCGGCCGCAGCCACCATGTCTAGAATGGTGAGCAAGGGCGAGG AGCTGTTC
**EP1201**	NNNACTAGTTCACTTGTCATCGTCATCCTTGTAATCGATGTCATG ATCTTTATAATCACCGTCATGGTCTTTGTAGTC CTTGTACAGCTCGTCCATGCCGAGAGTGATCC
**EP1429**	TTCAGGGTCAGCTTGCCGTAGG
**Ep1463**	GTGGGTTAGTGCGGCGGTTAGAC
**EP1499**	GGCTGGACGTAAACTCCTCTTC
**EP1622**	TACTTGGTCGGCAGAGCAGTTG
**EP1704**	CCCAACGAGAAGCGCGATCAC
**Nkx6-1 Crispr #11fwd**	CACCTCGCGCTCAGGACGAGCCCT
**Nkx6-1 Crispr #11 rev**	AAACAGGGCTCGTCCTGAGCGCGA
**Nkx6-1 Crispr #16fwd**	CACCCGTCCCACAGCCGGTTCCCC
**Nkx6-1 Crispr #16 rev**	AAACGGGGAACCGGCTGTGGGACG
